# Microglial Adenosine Receptors: From Preconditioning to Modulating the M1/M2 Balance in Activated Cells

**DOI:** 10.3390/cells10051124

**Published:** 2021-05-07

**Authors:** Rafael Franco, Alejandro Lillo, Rafael Rivas-Santisteban, Irene Reyes-Resina, Gemma Navarro

**Affiliations:** 1 CiberNed, Network Research Center, Neurodegenerative Diseases, Spanish National Health Institute Carlos III, 28034 Madrid, Spain; g.navarro@ub.edu; 2 Department of Biochemistry and Molecular Biomedicine, University of Barcelona, 08028 Barcelona, Spain; 3 Department of Biochemistry and Physiology, Faculty of Pharmacy and Food Science, University of Barcelona, 08028 Barcelona, Spain; alilloma55@gmail.com

**Keywords:** neurodegeneration, aging, Parkinson’s disease, Alzheimer’s disease, neuroprotection, neuronal survival, cannabinoids, receptor heteromers

## Abstract

Neuronal survival depends on the glia, that is, on the astroglial and microglial support. Neurons die and microglia are activated not only in neurodegenerative diseases but also in physiological aging. Activated microglia, once considered harmful, express two main phenotypes: the pro-inflammatory or M1, and the neuroprotective or M2. When neuroinflammation, i.e., microglial activation occurs, it is important to achieve a good M1/M2 balance, i.e., at some point M1 microglia must be skewed into M2 cells to impede chronic inflammation and to afford neuronal survival. G protein-coupled receptors in general and adenosine receptors in particular are potential targets for increasing the number of M2 cells. This article describes the mechanisms underlying microglial activation and analyzes whether these cells exposed to a first damaging event may be ready to be preconditioned to better react to exposure to more damaging events. Adenosine receptors are relevant due to their participation in preconditioning. They can also be overexpressed in activated microglial cells. The potential of adenosine receptors and complexes formed by adenosine receptors and cannabinoids as therapeutic targets to provide microglia-mediated neuroprotection is here discussed.

## 1. Introduction

Glial cells are key players in the functionality of the central nervous system (CNS). Astrocytes are more concerned with satisfying the energy and structural needs of neurons, while microglia have a surveillance function that mainly consists of preserving neurons from noxious events, but also of eliminating cell debris through phagocytosis. Astrocytes constitute a cellular target for neuroprotection [1,2], however, the focus of this review is microglia.

Microglia are considered immune cells that reside in the central nervous system (CNS). Microglial activation occurs in the development of the nervous system, in the healthy brain, and in a wide variety of circumstances, from cerebral hypoxia/ischemia to regions of neuronal death in neurodegenerative diseases. Experiments in human post-mortem samples show markers of microglial activation in an apparently healthy brain, that is, in individuals lacking clinical neurological symptoms. [3,4,5]. In ischemic stroke, the function of the activated microglia is complemented by activated macrophages infiltrating from the blood. In the case of, among others, epilepsy or neurodegenerative diseases, macrophages do not play any substantial role except in cases of impaired function of the blood-brain barrier. In all these cases, activation of the microglia/macrophages is considered neuroinflammation. Some authors prefer to speak of microglial activation and, eventually, pseudoinflammation [6], because the activation of the microglia is not necessarily associated with any pathology; for example, the developing brain is not considered inflamed. It should be noted that there is evidence of microglial activation as a consequence of lifestyle stress [7].

One of the first papers on ischemia and microglia described, in hippocampus, how activated microglia phagocytose degenerating neurons and express antigens of the major histocompatibility MHC-II complex [8]. While an acute traumatic event or stroke is likely to cause activation of microglia, activated microglia have been found in the brain or in patients suffering from neurodegenerative diseases [9], among others, from Parkinson’s disease (PD) [10,11], Alzheimer’s disease (AD) [12,13,14], and Huntington’s disease [15,16]. Addressing the exact role of microglia in these diseases is a challenge that has increased awareness of the potential of these cells because they can present two main phenotypes, known as M1 and M2, the first being pro-inflammatory and the second neuroprotective [2]. The gold standard in the field would be to find a way to convert M1 microglia to M2 with the ultimate goal of slowing the progression of neurodegenerative diseases [17]. Microglial activation was first assessed by phagocytic capacity, immunochemical studies, and by determining the release of pro-inflammatory cytokines. In the last two decades, new tools have been incorporated to characterize the microglial phenotype (M1, M2 and intermediate phenotypes) at the molecular level. These new tools are mainly taking into account the expression of proteins whose presence is abundant in one phenotype and scarce in the other (see [18]). At present, they are known as M1 or M2 markers and a significant number of them have been identified with commercial ad hoc antibodies already available to detect expression even in natural sources (such as brain slices).

Controversies have arisen related to the polarization of activated microglia, which have even led to denying the existence of M1 and M2 cells. [19]. It has been suggested that the phenotype of activated macrophages and microglia is constituted by a repertoire of cells with overlapping functions and markers [20]. However, the M1/M2 nomenclature has been and is, today, fundamental in both the macrophage and the microglia research fields. It should be noted that M2 macrophages or microglia can be further subdivided into 2a, 2b, 2c, and 2d (see [21,22,23,24,25,26] for review). As an example, a recent report shows that transplantation of M2-skewed microglia, produced upon interleukin-4 treatment, led to marked recovery of motor function in a model of spinal cord injury (SCI). Authors concluded: “*our results indicated that M2 microglia obtained by IL-4 stimulation may be a promising candidate for cell transplantation therapy for SCI*” [27].

G-protein-coupled receptors (GPCRs) modulate activation events in microglia. In this review, we have selected a subfamily of GPCRs, namely adenosine receptors (ARs) because they are relevant players in microglial function and because there are drugs targeting ARs that have recently been approved for the therapy of neurodegenerative diseases (see below).

## 2. Purinergic P1 and P2 Receptors

Purinergic nerves were discovered by late Prof. Geoffrey Burnstock, a truly inspiring scientist [28,29,30]. The purine nucleotide, ATP, may be released by different cells of the nervous system (see [31]); however, in some neurons it may be stored in vesicles and released upon a stimulus (see historical perspective in [32]). Apart from its action as neurotransmitter, ATP released to the extracellular medium exerts a variety of actions in every system of the human body. These actions are mediated by the so-called P2 purinergic receptors, which are located on the cell surface of the responding cell. There are two types of P2 receptors, those that are ligand-gated ion channels that are formed by homotrimers or heterotrimers of seven different subunits discovered so far (P2X_1_ to P2X_7_) [33] and GPCRs, known as P2Y, with eight members [34]. Virtually, any cell in the human body, for instance in the kidney [35] or in the lung [36], has one or more than one of those receptors. This paper does not focus on P2 but on P1 (or adenosine) receptors, which are those that recognize the nucleoside derivative, adenosine, produced after extracellular degradation of ATP. All adenosine receptors are expressed in the human brain but at various levels depending on the specific region (Table 1). Interestingly, the expression of mRNA transcripts for all types of adenosine receptors is elevated in the basal ganglia.

Extracellular ATP is degraded by ectonucleotidases to produce AMP that is the substrate of ecto-5′nucleotidase (CD73) whose reaction product is adenosine (extracellular). Intracellular adenosine participates in many metabolic processes. Indeed, adenosine can be released from cells to the extracellular milieu and, reciprocally, be uptaken to, eventually, resynthesize ATP via anabolic routes. There is also the possibility to convert extracellular adenosine into extracellular inosine by means of ecto-adenosine deaminase [37,38,39,40,41,42]. On the one hand, adenosine acts on P1 receptors located in neurons but is not released through synaptic vesicles; therefore, it is not considered a neurotransmitter but a neuromodulator. On the other hand, P1 receptors are expressed in virtually all cells of the human body, microglia included. 

P1 or adenosine receptors belong to the superfamily of G protein-coupled receptors (GPCRs). Four have been so far discovered: A_1_, A_2A_, A_2B_, and A_3_. A_1_ and A_3_ couple to Gi, thus inactivating the adenylate cyclase and decreasing intracellular cAMP levels. A_2A_ and A_2B_ couple to Gs, thus activating the adenylate cyclase and increasing intracellular cAMP levels. Therefore, adenosine receptor (AR) activation regulates protein kinase A activity (PKA). Moreover, PKC may be activated via A_2B_ receptor-mediated intracellular calcium mobilization [43,44,45], and other pathways may be also affected, e.g., the mitogen activated protein kinase (MAPK) pathway. Finally, ion fluxes are differentially affected by adenosine acting on ARs [46]. It should be noted that GPCRs may interact leading to heteromers whose function is different from that of the interacting receptors [47]. There are several examples of heteromers formed by ARs; they may interact with each other, for instance, to form A_1_-A_2A_ and [48,49,50,51] A_2A_-A_2B_ complexes [52,53], or with other members of the GPCR superfamily, for instance to form A_2A_-CB_1_ or A_2A_-CB_2_ complexes [54,55,56]. There is solid evidence on the relevance of AR-containing heteromers for the modulation of microglial activation and as therapeutic targets to combat neurodegenerative diseases [57].

Mitochondria homeostasis in neurons appears as a key factor in preventing neurodegeneration [58]. Despite GPCRs are thought to act in response to extracellular stimuli, they can be also found in mitochondria [59,60,61], where they may participate in the control of oxidative burden and mitochondrial performance. The future will tell whether GPCRs in neuronal and / or glial mitochondria can be therapeutic targets to combat neurodegenerative diseases. Interestingly, the risk of neurodegenerative diseases is reduced after consuming natural adenosine receptor antagonists, namely theophylline (tea) and caffeine (coffee and cola drinks) [62,63,64,65,66,67,68,69,70,71,72,73,74,75,76,77].

## 3. Potential of Adenosine Receptors (AR) as Therapeutic Targets

After several inconveniences in the race to obtain new drugs that act on ARs, there is evidence of excellent prospects for the approval of the human use of ligands of these receptors.

For many years, adenosine itself was the only drug targeting ARs that was approved for human use. Despite the early discovery of the actions of adenosine in the cardiovascular system [78], to our knowledge, there are no AR-related drugs in the line to combat cardiovascular disease. However, adenosine has saved lives in the emergency room as it converts paroxysmal tachycardia into sinus rhythm. The main basis for proposing this intervention, only performed at hospitals, was the work performed in the second half of the fifties by different laboratories. The data from Berne’s laboratory allowed patenting the use of the nucleoside for combating tachycardia [79,80,81]. It is intriguing why there are no new drugs targeting adenosine receptors able to combat heart diseases, especially after the finding that adenosine A_2A_ receptor (A_2A_R) antagonists, which are safe, are efficacious in reverting abnormal calcium handling in cells from patients with atrial fibrillation [82,83,84], a disease lacking efficacious medication.

In general, AR antagonists are safe. The most consumed (worldwide) psychoactive compounds are AR antagonists. We refer to natural methylxanthines, e.g., caffeine in coffee, theophylline in tea and theobromine in cocoa. Those methylxanthines are considered as generally safe [76,85,86,87]. They have been approved for human use; they are present in a variety of OTC (over-the-counter) medications and in some therapies of respiratory diseases. In addition, consumption of methylxanthines decreases the risk of suffering from neurodegenerative diseases, whose main risk factor is age [67,71,72,76,86,88,89]. 

A_2A_R antagonists have been developed in parallel in different pharmaceutical companies. They were designed to enter the brain and be effective for Parkinson’s disease (PD), a neurodegenerative disease that involves the degeneration of dopamine-producing neurons in the substantia nigra. Due to the opposite dopamine-adenosine functionality in the striatum, it was hypothesized that the action of dopamine in PD patients could be enhanced if the A_2A_R was blocked [90,91,92,93,94,95,96]. Furthermore, experiments in animal models suggested that blockade of the A_2A_R affords neuroprotection, thus raising the possibility that AR antagonists delay the progression of this neurodegenerative disease [74,75,97,98,99,100,101,102,103,104,105,106,107]. Highly selective A_2A_R antagonists were developed and few years ago the first-in-class drug was approved for coadjuvant therapy in PD. It was KW-6002, also known as istradefylline (PD) [108,109] that was first approved in Japan (Nouriast^TM^) and years later, in the USA (Nourianz^TM^). Such decisions by regulatory bodies in two different and populated countries pave the way for approval of AR ligands for a variety of diseases. Even in cancer, there is great hope because AR antagonists improve the efficacy of immunotherapies [110,111,112,113,114].

## 4. Neuron vs. Glia in Neurodegeneration

A fundamental question to address in the field of neuroprotection is whether to target neurons or glia. In our opinion, neurons have been at the center of the stage to explore and test neuroprotective interventions to slow the progression of neurodegenerative diseases. However, a direct action on neurons is challenged by the poor survival prospects of neurons that are having problems and will, sooner or later, die. In fact, blocking a presumed death mechanism in neurons may not be effective for senescent or dysfunctional neurons [115]. Cell therapy may overcome such problem as neuroprotection consisting in increasing the number of cells without necessarily affecting the fate of existing neurons. On the contrary, it is doubtful that gene therapy with viral vectors, aimed at infecting suffering neurons, can prevent neurodegeneration: it may help reduce symptoms, but there is no reason to believe that gene therapy can increase the lifespan of an infected neuron.

Neurons require glia to survive and maintain proper functionality. Glial cells can certainly help accelerate cell death, but they are effective in preventing or delaying neuronal death [115]. It is well known that astroglia exchange regulatory molecules with neurons to which they also provide molecules necessary for energy production. Neuron-microglia interaction is less evident under homeostatic conditions. However, these interactions play an important role in cerebral hypoxia/ischemia and neurological diseases associated with inflammation. In addition, the functionality of the microglia is essential in physiological neuronal death, which occurs both in the development of the nervous system and later in human life. Neuron-microglia interactions have two sides, one related to the removal of neuronal components after death and the other aimed at both starting and stopping inflammation. In summary, the glia seems a better target than neurons to provide neuroprotection. In keeping with the title of the special issue in which this article is included, we will address the potential of microglia to protect neurons and/or provide neuroprotection through proper manipulation of the M1/M2 phenotypic balance (see below).

## 5. Microglia

Microglia are considered as part of the immune system located in the CNS. Their role is similar to that of blood macrophages, which are characterized by two functions, phagocytic and inflammatory. Microglial cells were identified by Pio del Rio Hortega, a contemporary of Santiago Ramón y Cajal [116,117,118]. Activation of microglia was, for several years, considered detrimental; activated cells were described as reactive microglia (see [119] for review). It is now known that these cells are important for neuroprotection and the reason is that there are different phenotypes resulting from microglial activation [11].

Macrophages are key in the fight against a variety of infections of parasitic, fungal, bacterial and viral origin. From a resting state, they undergo activation to display a M1, or proinflammatory phenotype, or follow an alternative activation route leading to the so-called M2 phenotype, which participates in resolution of inflammation and cleanup. The properties of the two populations in the context of a bacterial infection were concisely described in [120]:

*“Based on limited numbers of markers, activated macrophages can be classified as classically activated (M1) macrophages that support microbicidal activity or alternatively activated (M2) macrophages that are not competent to eliminate pathogens”.*

Ontogenesis and anatomical studies led to i) recognize microglia as resident cells in the CNS, ii) recognize that these resident cells may activate, and iii) major lesions may lead to the entrance and activation of macrophages from blood [121,122]. 

Under homeostatic conditions, the microglia are at rest (M0). Any damaging condition results in cell activation that, analogously to macrophages, may lead to different microglial phenotypes. As reviewed elsewhere, the main activation phenotypes are M1 and M2, although the M2, depending on the specific function and the markers that are expressed, may be subdivided into 2a, 2b, 2c, and 2d [18]. 

**Figure 1:**
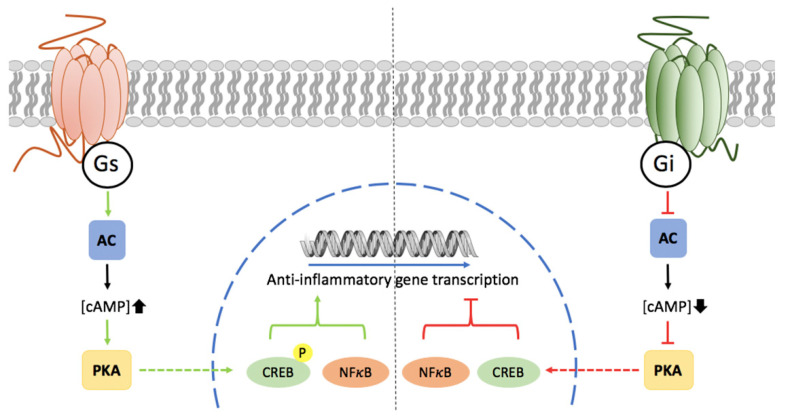
Cell surface GPCRs and the pCREB pathway of transcription regulation of genes producing anti-inflammatory mediators in microglia. Gs-coupled GPCR activation increases cAMP production, activates protein kinase A (PKA), and phosphorylates the cAMP response element-binding (CREB) that induces the transcription of genes related to anti-inflammatory processes. Conversely, Gi-coupled GPCR activation leads to inactivation of the p-CREB pathway. Green color means activation/potentiation and red color means deactivation/blockade.

**Figure 2:**
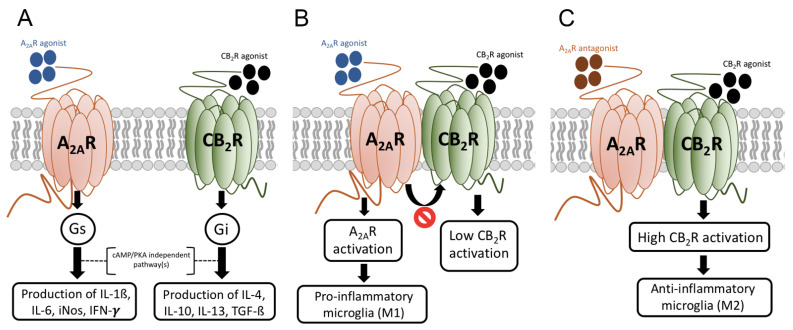
Microglial polarization mediated by adenosine A_2A_ and cannabinoid CB_2_ receptor and by A_2A_-CB_2_ receptor heteromer. Panel (**A**). By unknown mechanisms unrelated to the cAMP/PKA pathway activation of the A_2A_ receptor, which couples to Gs, this receptor mediates pro-inflammation, whereas activation of the CB_2_R mediates anti-inflammation/neuroprotection. Panel (**B**). A_2A_R agonists acting on microglial receptors are pro-inflammatory. Within the A_2A_-CB_2_ receptor heteromer, activation of the A_2A_R reduces CB_2_R-mediated signaling (negative cross-talk). Panel (**C**). Blockade of the A_2A_R is anti-inflammatory in activated microglia but, also, it reverts the negative cross-talk within the A_2A_-CB_2_ receptor heteromers. Accordingly, A_2A_R antagonists are not only anti-inflammatory but potentiate the anti-inflammatory/neuroprotective action of endocannabinoids acting on microglial CB_2_Rs.

GPCRs are involved in the regulation of microglial polarization. Actually, important clues related to microglial polarization come from detailed studies on how neuropeptides inhibit classical microglial activation thus suggesting that they may induce M2 polarization. The actions of the vasoactive intestinal peptide (VIP) on reducing microglial production of pro-inflammatory cytokines are due to activation of vasoactive intestinal peptide receptors 1 and 2 (VPAC1 and VPAC2) [123,124]. Neuroprotection by VIP acting on microglial receptors may be due to IL-4 production and protection of hippocampal neural stem/progenitor cells [125]. Pathways engaged upon GPCR activation can regulate microglial activation and polarization. Gs coupling and PKA pathway activation impacts on NFKB transcriptional activity thus inhibiting chemokine gene expression. Expression of complexes formed by CREB binding protein (CBP) and NFKB may be regulated via GPCRs (Delgado, 2002). Accordingly, GPCRs via Gs/Gi, i.e., via modification of cAMP levels, modulate microglial activation by balancing the action of these transcription factors (Figure 1) [126,127]. Neuropeptides acting via Gs/cAMP/PKA inhibit MAPK4, impact on the JNK pathway and on the composition of cJun/cFos and cJunB complexes, thus reducing the expression of IFN-gamma, CD40, CXCL10 and iNOS [126,128]. However, not all Gs-coupled receptors in microglia mediate neuroprotection, adenosine A_2A_ receptor activation increases the expression of nitric oxide in microglia [129] while cannabinoid receptors mediate neuroprotection despite they are coupled to Gi. This means that there are different pathways that impact on the final output in terms of production of pro-inflammatory or anti-inflammatory mediators (Figure 2A). It would be very interesting to study the time course variations in the activation program of different pathways. Consistent with differences in protein expression / functionality in microglia throughout the course of inflammation, there are GPCRs expressed at low levels in resting microglia but overexpressed upon activation. A_2A_ receptors are one example, they are barely expressed in resting microglia, but are markedly upregulated in surrounding microglial plaques found in AD patients [130]. Interestingly, the adenosine A_1_ receptor is also up-regulated in neurodegenerative structures in AD and its activation modulates both phosphorylation and translocation of tau and processing of the amyloid precursor protein [130].

There is a kind of controversy surrounding the term “neuroinflammation” because it is doubtful that CNS becomes inflamed. Moreover, the function of activated microglia in CNS development is not considered to result in neuroinflammation. Therefore, it is suggested that neuroinflammation should be substituted by microglial activation or CNS pseudoinflammation [6]. In fact, microglia become activated in physiological/healthy CNS development. If neuronal death occurs (i) occasionally throughout human life and (ii) progressively in healthy-aged individuals, microglia are likely to be activated. In various pathological conditions, including neurodegenerative diseases, the microglia are activated. When should microglial activation be considered inflammation? What if some type of microglial activation is needed for neuronal survival in both health and disease? Moreover, neuronal death requires the removal of that debris by the phagocytic activity of activated microglia. Undoubtedly, the overproduction of pro-inflammatory cytokines when there is an imbalance in the M1/M2 ratio can lead to further neuronal death. In summary, microglial activation is a physiological mechanism that can become dangerous and potentiate certain neuropathology if the skewing towards the M2 phenotype does not occur in the appropriate period of time.

Although the expression of ARs depends on the state of the microglia (resting or activated) and the specific phenotype, all ARs, except the A_2B_, have been reported to be expressed in resting cells. [131,132]. Expression may vary depending on microglial location in the brain. It is likely that the A_2B_ receptor is expressed in M1 and/or M2 skewed cells. In fact the A_2B_R is present in primary microglia from rat forebrain and its activation (in resting cells) engages the p38 MAPK pathway to induce interleukin(IL)-6 release [133].

After an excitotoxic insult in cortex or striatum, A_2A_R antagonists differentially modulate astrogliosis and microglia activation. In microglia activated upon quisqualic acid-induced excitotoxicity A_2A_R antagonists inhibit the expression of cyclooxygenase-2 (COX-2) [134]. On the other hand, excitotoxicity by glutamate activates glutamate N-Methyl-D-Aspartate (NMDA) receptors expressed in microglia and leads to the release of pro-inflammatory cytokines [135]. A vicious circle sustaining M1 microglia and neuronal cell death may be established unless any physiological action restoring homeostasis or any pharmacological intervention. For instance, targeting adenosine receptors leads to M2 skewing. NMDA receptor function in microglia is increased by direct interactions with A_2A_Rs, increasing the possibility that A_2A_R antagonists may be neuroprotective by reducing the excitotoxic load associated with neurodegenerative diseases [136].

It is well known that the development of the nervous system requires the programmed death of a significant number of neurons [137]. Less well known is that neuronal death is a lifelong physiological process. Indeed, individuals with epileptic seizures do loss neurons in each episode [138,139]. But, also, healthy individuals seemingly lose neurons upon aging. Fortunately, loss of neurons per se does not lead to disease, either because of the redundancy in neural circuits or because neuronal death is not focused on a specific region. Redundancy is also observed in the motor control circuits of the basal ganglia, since it is estimated that clinical symptoms in parkinsonian patients appear when the number of nigral cells lost is 70%.

Age is the main risk factor in the most prevalent CNS neurodegenerative diseases, Parkinson’s and Alzheimer’s. Accordingly, the progressive loss of neurons in the CNS of the aged human does not lead to disease in physiological aging but may lead to neurodegenerative diseases for which no cure exists. In fact, there are few and non-optimal therapies to combat Alzheimer’s or Huntington’s diseases. In the case of parkinsonism, the work and wisdom of Hornykiewicz and colleagues allowed detection of a loss of dopamine in certain brain areas of the patients. They noticed the poor brain penetrance of dopamine and suggested a treatment with the precursor of the neurotransmitter, levodopa (L-DOPA). L-DOPA is able to cross the blood–brain barrier and is readily processed to dopamine in the CNS [140,141,142,143,144]. L-DOPA is still used today to treat PD symptoms but, unfortunately, it does not delay disease progression. The issue is, therefore, how to afford neuroprotection in neurodegenerative diseases and, eventually, in long-lived healthy individuals. Here we will discuss how microglia may have a neuroprotective role in both physiological and pathological aging. The difficulties in demonstrating the efficacy of neuroprotection interventions in humans (see [75]) is a hot topic whose discussion is out of the scope of the present paper.

## 6. Ischemic Preconditioning after Brain Ischemia 

Preconditioning is a mechanism by which exposure to an insult prepares the whole system to better respond to a second similar insult. To our knowledge it was first discovered in the cardiovascular system. Upon survival a heart infarction the cardiovascular system is better suited to respond to a second one. This preconditioning is mechanistically complex but adenosine receptors (AR) are key players. This is probably due to the fact that in glucose and/or oxygen deprivation, ATP is readily converted into adenosine, whose concentration increases in the blood and in any (local) extracellular environment. The first results linking AR to preconditioning in the ischemic (rabbit) heart appeared in the nineties [145]. The A_1_ type was presented as the most important receptor in preconditioning [146] but this was probably due to neglecting for decades the relevant role of other AR types in heart function. In a model of ischemia-reperfusion the synergistic action of A_1_R and A_2A_R agonists on cardioprotection was reported in 2010 [147]. 

Ischemic preconditioning in the cardiovascular system prompted scientist to focus on the occurrence of a similar mechanism in the ischemic brain. The protection of hippocampal cell death afforded by sub-lethal ischemia is among the earlier finding in this issue [148]. Soon afterwards, it was reported that ARs were involved in the preconditioning mechanisms [149,150,151,152].

The question relevant for the present article is whether microglial ARs play a role in preconditioning. First of all it was soon known that both microglia and astroglia play a significant role in ischemic preconditioning [153,154]. Despite the relevant role of AR in modulating microglial function, studies aimed at answering the question of involvement of microglial AR in preconditioning after brain hypoxia are scarce [131,155]. Either microglial ARs are not important for brain ischemic preconditioning or work related to the ischemic brain has focused on neurons, as the focus in the ischemic heart was placed on cardiomyocytes. In brain ischemia-reperfusion injury, the neuroprotective role of targeting ARs has been demonstrated, although activation of the A_1_R receptor is neuroprotective, drugs that activate these receptors have cardiovascular side effects; thus the alternative consists of blocking the effect induced by A_2A_R through the use of antagonists that, in general, are very safe [156]. In addition, the expression of ARs may be modified after an ischemic insult [105,157,158,159,160,161,162]. In summary, it is likely that ARs are relevant for the functionality and fate of microglia that become activated in ischemia [97,163,164,165,166,167]. Abbracchio and Cattabeni, already in 1999, suggested that antagonists of the A_2A_R could be useful in neuroprotection by both reducing the neuronal release of glutamate, an excitatory neurotransmitter, and to regulate the activation of microglial cells [105]. 

## 7. Microglia in Aging and in Neurodegenerative Diseases, Friend or Foe?

Microglia are instrumental in the events causing neuronal death during the development of the nervous system and, also, in the clean-up after such neuronal death. It would be naïve to think that, in the absence of any event resulting in clinical symptoms, i.e., in a lifelong physiological/healthy brain, microglia remain static. 

Although the data are scarce, neuronal death occurs throughout the individual’s life, although at a much slower rate than during the development of the nervous system. A seminal review in 2007 [168] highlights that the cross-talk between microglia and neurons in developmental stages encompass, among other, Purkinje cell death via microglia-induced respiratory burst, release by microglia of factors that lead to neuronal apoptosis and microglia-induced synaptogenesis and synaptic properties. The role of microglia on maintaining CNS homeostasis in a healthy brain is less know. In words of Graeber, it refers to microglia as: “*analogous to electricians, they are capable of removing defunct axon terminals, thereby helping neuronal connections to stay intact*” [169]. Apart from the role in removing cells that are targeted to die along development, the hypothesis is that, in adult stages, microglia help removing cells that are targeted to die, e.g., those that are not very active and die, to reinforce the synaptic connections of the surviving cells and firm up those neural circuits that seem more necessary. 

The evaluation of neuronal death is usually aimed at detecting an underlying pathology. In our opinion, this should be questioned, as neuronal death cannot be ruled out in an apparently healthy brain. It is tempting to speculate that physiological aging correlates with neuronal loss but strengthening the synaptic connections that the individual most needs in their daily life. A few years ago it was noted that neurons can die in several ways: “*intrinsic and extrinsic apoptosis, oncosis, necroptosis, parthanatos, ferroptosis, sarmoptosis, autophagic cell death, autosis, autolysis, paraptosis, pyroptosis, phagoptosis, and mitochondrial permeability transition*” [115]. It is likely that some of those may be operating in the brain of a healthy aged individual, i.e., not only in patients suffering from neurodegenerative diseases or in patients suffering from a stroke. Despite the difficulties in assessing neuronal death and neuron-microglia cross-talk in the adult brain, future work is required to confirm the bidirectional interactions and to decipher the underlying mechanisms.

## 8. Skewing the M1/M2 Balance towards the Neuroprotective M2 Phenotype

The real state of microglia in the aged brain is not fully elucidated. However, it is suggested that senescent microglia may contribute to age-related neurological diseases. The reduction of phagocytosis in senescent microglia probably prevents the adequate elimination of debris and the predisposition to be activated through the M1 pathway, while the difficulty to develop an M2 phenotype may impede the physiological function of protecting neurons from death [170,171,172]. In any case, avoiding the senescence in microglia appears as a good strategy to decrease the risk of neurodegenerative diseases. In other words, reduced microglia senescence may underlie a physiological aging. It should be noted that a portion of microglial cells are activated in physiological aging. In fact, using a marker of activated microglia, (R)-[^11^C]PK11195, positron emission tomography (PET) brain scans of healthy subjects aged 19 to 79 showed an increased activation upon aging. Authors conclude that “*activated microglia appear in several cortical and subcortical areas during healthy aging, suggesting widespread neuronal loss*” [173].

Working on the expression and function of cannabinoid receptors in resting and activated microglia we found that expression of cannabinoid CB_1_ and CB_2_ receptors in microglia (resting) from a transgenic rodent model of AD was similar to that observed upon activation of microglia from wild type mice. As the cognition deficits in AD animal models are only evident upon aging, it was tempting to speculate that a certain degree of chronic activation was neuroprotective. It is assumed that such activation is constituted by cells skewed to the M2 phenotype [174]. 

GPCR function is modulated by interaction with other members of the superfamily. We have found interesting results with cannabinoid receptors. There are two types of cannabinoid receptors, CB_1_ and CB_2_, and both are capable to interact with AR. In microglia, the A_2A_R may directly interact with the CB_2_R and the structure of the resulting complex is such that the blockade of the A_2A_R by a selective antagonist increases signaling through CB_2_R [54]. A_2A_R antagonists, appear, once more, as beneficial; in this case by increasing the action of a receptor that, expressed in glial cells, is considered to be neuroprotective [175,176,177]. In fact, cannabinoid receptors are now considered promising therapeutic targets for fighting neurodegenerative diseases [178,179,180]. A review on the role of A_2A_R-containing heteromers in neurodegenerative events and in microglia activation is provided in [57]. 

The A_2A_R regulates several functions derived from microglial activation. First of all, A_2A_R activation modulates microglial motility [181]. Furthermore, in mixed glial cultures (astrocytes/microglia) we found that activation of the A_2A_R results in potentiating the release of nitric oxide by activated microglia. The effect was dependent on the presence of astroglia although both A_2A_R expression and NO synthase-II immunoreactivity were only observed in microglia. These actions, which were not detected in cocultures obtained from A_2A_R KO animals, suggest that the neuroprotection provided by A_2A_R blockade comes, at least in part, from effects mediated by receptors expressed in activated microglia [129]. Another action of A_2A_R antagonists results from negative crosstalk when A_2A_ and CB_2_ receptors are expressed as heteromers [54,182]. By interprotomer interactions within the heteromer, activation of A_2A_R partially blocks CB_2_R-mediated signaling, which in microglia leads to the production of neuroprotective factors. Therefore, blocking A_2A_R would reduce the expression of pro-inflammatory mediators (via the A_2A_R) and release the brake for CB_2_R activation, leading to the production of neuroprotective molecules (Figure 2B). Studies in the hippocampus also identified A_2A_R as modulating the recruitment and activation of microglia [102]. In experiments performed in a microglial cell line A_2A_R antagonists decrease proliferation of activated microglia and the release by these cells of brain-derived neurotrophic factor (BDNF) [182]. A review on the potential of targeting microglial A_2A_R to combat neurodegenerative diseases is found in [183]. Other AR types may participate in adjusting the activation of microglia related to neurodegenerative diseases but they seem of less relevance than the A_2A_R. Whereas the A_3_R is expressed in microglial cells [184], a recent paper shows the action of A_2A_R antagonists and A_1_R agonists on the production of pro-inflammatory cytokines [185]. What is now necessary is to address the expression of AR types in resting, and in activated M1 and M2 microglia and to address the mechanisms of skewing to the M2 phenotype targeting AR and AR-containing heteromers.

## Figures and Tables

**Table 1:** Comparative expression of mRNA transcripts of adenosine receptor in different regions of human brain.

	mRNA Transcript Expression Levels (Scaled Tags Per Million)											
	Cerebral Cortex	Olfactory Region	Hippocampal Formation	Amygdala	Basal Ganglia	Thalamus	Midbrain	Pons and Medula	Cerebellum	Corpus Callosum	Spinal Cord	Pituitary Gland
**A_1_R**	139.6	83.6	92.2	79.4	149.2	100.7	139.3	143.9	74.2	147.8	100.7	2.7
**A_2A_R**	9.4	3.6	5.8	3.9	53.4	13.7	3.0	6.0	3.0	3.6	0.8	1.1
**A_2B_R**	14.3	8.9	14.5	12.8	15.5	4.9	0.9	11.3	13.7	3.9	7.9	1.9
**A_3_R**	27.3	9.9	31.9	33.9	47.9	47.9	61.3	50.9	6.6	32.6	98.4	7.1

Data taken from human brain protein atlas using FANTOM5 dataset and Cap Analysis of Gene Expression (CAGE). Data can be found in https://www.proteinatlas.org/search/adenosine+receptor (Accessed on 12 April 2021). For each receptor, a color scale shows higher (darker) versus lower (lighter) expression; the highest expression level for each receptor is underlined.

## Data Availability

Data in Table 1 is directly retrievable from https://www.proteinatlas.org/search/adenosine+receptor (Accessed on 12 April 2021).
